# The Examiners Meeting

**Published:** 1899-08

**Authors:** 


### The Examiners Meeting.

The National Association of State Dental Examiners held their annual meeting on July 29th to 31st. We regret to state that not as many State Boards were represented as ought to have been, nevertheless the organization accomplished its usual work. A joint meeting was held by a committee from this body with a like committee from the National Association of Dental Faculties and action was taken, which resulted in the clearing away somewhat of a misunderstanding and friction, that has existed between these bodies for several years. This is a result that should be hailed with satisfaction by every member of these two bodies, and indeed by all dentists who take an interest in the welfare of the profession. Doubtless in the past there has been friction, for which both parties were in some degree responsible. These difficulties seem now to be entirely removed, and the two bodies will work in harmony, so that jointly they will work for the strengthening and building up of the profession. This was the object of the organization of these two bodies, that they should co-operate in this great work. They have arrived at an understanding now by which the original aim and intent will be carried out.

May the seeds of discord never again be sown, but harmony rule always.

